# Effects of Harpin and Flg22 on Growth Enhancement and Pathogen Defense in *Cannabis sativa* Seedlings

**DOI:** 10.3390/plants11091178

**Published:** 2022-04-27

**Authors:** Lauren B. Sands, Taylor Cheek, Joseph Reynolds, Yi Ma, Gerald A. Berkowitz

**Affiliations:** Agricultural Biotechnology Laboratory, Department of Plant Science and Landscape Architecture, University of Connecticut, Storrs, CT 06269, USA; lauren.sands@uconn.edu (L.B.S.); taylor.cheek@uconn.edu (T.C.); joseph.d.reynolds@uconn.edu (J.R.)

**Keywords:** *Cannabis sativa*, PAMP, harpin, flg22, hemp, *Pythium*, ethylene signaling

## Abstract

Pathogen-associated molecular patterns, PAMPs, are a diverse group of molecules associated with pathogenic microbes and are known to activate immune response and in some cases enhance growth in plants. Two PAMPs, harpin and flg22, have shown these affects in various plant species. PAMPs are known to activate basal immunity, the ethylene signaling pathway, alter gene expression and change plant composition. Pretreatment with harpin enhanced hemp seedling resistance to *Pythium aphanidermatum*, while flg22 failed to induce the defense mechanism towards *P. aphanidermatum*. In the absence of the pathogen, both harpin and flg22 enhanced seedling growth when compared to the water control. Ethylene is a hormone involved in both plant defense signaling and growth. Both harpin and flg22 pretreatment induced certain ethylene responsive genes but not all the genes examined, indicating that harpin and flg22 act differently in ethylene and potentially defense signaling. In addition, both harpin and flg22 induced *CsFRK1* and *CsPR1*, two marker genes for plant innate immunity. Both PAMPs can enhance growth but likely induce different defense signaling pathways.

## 1. Introduction

*Cannabis sativa* is an herbaceous crop with many economical and medicinal benefits. Understanding cannabis’s internal mechanisms for growth and homeostasis is very important for the industry. A range of reports claim that exposing plants to Pathogen-Associated Molecular Patterns (PAMPs) can enhance growth development and improve disease resistance. PAMPs are molecular structures originating from a vast array of microbes and have been known to activate immune defense in plants [1]. Administering PAMPs to various plants has a history of increasing plant growth, germination rate, and disease resistance [1,2,3,4].

Mature plants and young seedlings treated with PAMPs have shown changes in development and defense responses. Such studies involve plant species such as *Arabidopsis thaliana* [2], *Vitis vinifera* (grape) [5], and *Solanum lycopersicum* (tomato) [6]. When PAMPs are introduced to plants, plant defense is triggered, regardless if there is an actual pathogen present. Pattern Recognition Receptors (PRRs), localized on the plant cell plasma membrane, recognize PAMPs and initiate PAMP-triggered immunity (PTI) [7], which allows plants to protect themselves. A few of the intracellular responses related to PTI include rapid ion fluxes across the plasma membrane, production of reactive-oxygen species, and rapid changes in gene expression and cell wall enforcement [8].

Flagellin 22 (flg22), a well characterized PAMP, is a 22-amino-acid peptide from the bacterial protein, flagellin [9,10]. Flg22 has been known to induce PTI in plants when administered, resulting in ethylene biosynthesis, activation of mitogen-activated protein kinase cascades, and changes in gene expression [4].

Harpins form a family of proteins and originate from various organisms. Harpin_EA_, the protein used in our study, originates from the Gram-negative bacterium, *Erwinia amylovora*. *E. amylovora* causes fire blight in the Rosaceae family of vegetables [11]. Harpin has been touted anecdotally as a cannabis growth promoter and disease resistance aid; however, there have been no publications supporting this assertation. Following application to plants, harpin is thought to induce insect defense, activate the ethylene signaling pathway, and increase photosynthetic rates [2,3]. A common functionality seen between flg22 and harpin is that both are thought to activate the ethylene signaling pathway [3,4]. Ethylene can regulate plant defense responses to both biotic and abiotic stresses [12].

In this report, we used *Pythium aphanidermatum* as the pathogenic source. *Pythium* species are soil-borne plant pathogens classified as oomycetes [13]. They often cause damping-off and *P. aphanidermatum* is known to be very aggressive towards plants in their germination and seedling stages [14,15], hence the use of cannabis in the seedling stage in this report.

Pathogen-defensive and ethylene-responsive genes, shown in Table 1, were monitored in cannabis seedlings following PAMP incubation. The effects of flg22 and harpin on indicated genes were compared in the assays to gain a better understanding of how signaling pathways are affected. PAMPs collectively are understood to enhance growth and defense toward pathogen infection. While there are countless publications regarding PAMP interactions with other plant species, there are none regarding cannabis. There is a lack of publications and experimental evidence supporting the use of PAMPs in cannabis. This study examines defense response, gene expression, and growth enhancement to further understand how flg22 and harpin affect cannabis.

## 2. Materials and Methods

### 2.1. Plant and Microbe Materials

Cannabis seedlings used were a commercially sourced fiber hemp variety. The original name of the strain was unknown, therefore, plant tissue was sent for genotype testing at Phylos^®^. Phylos^®^ is a company based in Oregon that performed genotype testing on strains of cannabis. The seeds were grown out to full vegetative size under a 16 h light/8 h dark photoperiod. Plant tissue was prepared as instructed by the company. The results of the genotype indicated that the variety is closest to Finola Hemp; therefore, we named it Finola2 (Figure S1). Finola is a fibrous strain, oftentimes used to produce hemp oil [22]. It was also found out that Finola2 has a moderate level of genetic variation, low levels indicate homozygosity and true breeding, while high levels indicate high heterozygosity without true breeding. Therefore, this strain has a moderate level of true breeding potential and will not produce offspring with too much variety of physical characteristics.

The *Pythium* strain used in this study is *P. aphanidermatum* KOP8, collected from microgreens (wheatgrass) from Kroppert Cress in NY. The sample was isolated from seeds in July 2013 and the accession number on NCBI blast is #MG993547.

### 2.2. Pathogenicity Test

Seeds were separated into two piles: dark colored, viable seeds, versus light colored, non-viable seeds. Seeds that are darker and more uniform often indicate higher germination success [23]. Viable seeds were then placed into autoclaved cheese cloths and secured using zip-ties. Seeds were surface sterilized with bleach and ethanol in a sterile hood. The bags were submerged into a 70% ethanol solution for 2 min, into a 3% bleach solution for 20 min, then washed with DI water [24]. Seeds were then imbibed in either DI water, 0.5 g/L harpin (*Axiom*, Rx Green Technologies), or a 100 μM flg22 solutions for 48 h in the dark.

The *P. aphanidermatum* originated from a stock mycelial plate, which was incubated in the dark at room temperature until needed. To obtain fresh mycelia, an 8 mm plug was taken from the stock pythium agar and placed on corn meal agar Petri plates (Fisher Scientific, Hampton, NH, USA). The pythium grew for 3 d in the dark at room temperature and the fresh mycelial growth was used for inoculation [25].

After 48 h of imbibition in PAMP solution, seeds were removed from the boxes and spread out on sterile paper towels. Using flame-sterilized forceps, seed coats were removed, leaving the seed embryos. The embryos were placed onto Petri dishes containing water agar. Five seeds were placed on each plate, 1 cm from the edge in circle formation. An 8 mm plug of agar containing pythium mycelia was placed directly in the center of the plates containing the seeds [14]. There were three treatments: water soak with pythium, harpin soak with pythium, and flg22 soak with pythium, with a total of 20 seeds per treatment. The water treatment was the control. The plates were sealed using parafilm and incubated in the dark at room temperature for 7 d, in randomized placement design, allowing seedlings to grow and become infected with pythium. The experiment was repeated twice.

Seeds were removed individually, and each length was measured. The length was measured in centimeters for each seedling. Each plate comprised one biological replicate (five seedlings each, as shown in Figure S2). The biological replicates’ measurements were averaged and separately compared to the control for an analysis of variance.

### 2.3. Gene Expression Analysis

Viable seeds were surface sterilized, then imbibed in water, harpin or flg22 solutions containing the same concentrations as indicated previously. These seeds were imbibed in the dark for 24 h, then seed coats were removed in sterile conditions. The seed embryos were placed onto water agar Petri dishes (five seeds per plate), allowing the seedlings to grow for either 24 or 48 h post treatment [26]. Posttreatment time was determined by the time of seed removal from the PAMP solution. There were four plates per each of the three treatments for each time point (24- and 48-h post treatment), with a total of 24 plates. The plates were incubated in the dark in a randomized placement design.

Following PAMP incubation, seedlings were collected, and flash frozen in liquid nitrogen. Total RNA was isolated from the seedlings using Plant and Fungi RNA Extraction Kit (Macherey Nagel, Duren, Germany). cDNA was synthesized using iScript Reverse Transcriptase (Bio-Rad, Hercules, CA, USA).

qPCR was performed using iTaq Universal SYBR Green Supermix (Bio-Rad). Primers are listed in Supplemental Table S1. The qPCR was run in a CFX Connect Real-Time PCR System (Bio-Rad). *CsUbiquitin10* was used as a housekeeping gene. Cannabis *ERF1*, *EIN2*, *EIN5*, *PR1* and *FRK1* genes were identified using homologous *Arabidopsis thaliana* genes. Four biological replicates were analyzed. Primers are listed in Table S1.

### 2.4. Growth Test

The protocol for seed sterilization, seed imbibition, and seed coat removal remains the same. Five seed embryos were placed onto their respective Petri plate containing water agar, equidistant apart and about 1 cm from the edge of the plate. There were three treatments, water, harpin, and flg22, and a total of 25 seeds per each treatment. The plates containing the seeds were closed using parafilm, then incubated at room temperature (~22 °C) in the dark in a randomized placement design for 7 d. At the end of the incubation period, the Petri plates were removed from incubation and opened. Seeds were removed individually, and each length was measured. Each plate comprised one biological replicate (five seedlings each, as shown in Figure S2). The measurements of the biological replicates were averaged and separately compared to the control for an analysis of variance.

## 3. Results

### 3.1. Harpin Induces Resistance to P. aphanidermatum in Hemp Seedlings

**Figure 1:**
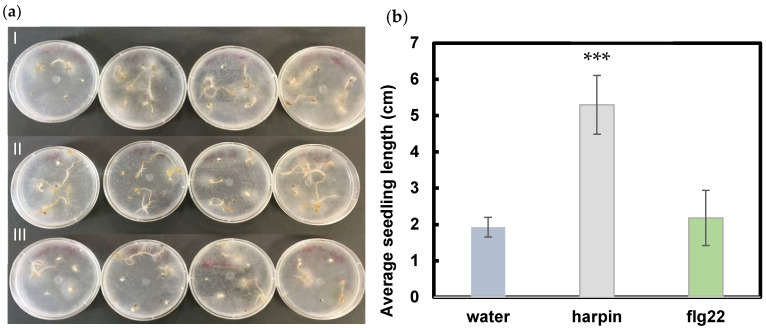
Effects of harpin and flg22 on seedling growth in the presence of *P. aphanidermatum*. (**a**) Seedlings grown on agar plates for 7 dpi with *P. aphanidermatum*. I. water control. II. harpin. III. flg22. All four biological replicates for each treatment, of one experimental replication, are shown. (**b**) Average length of seedlings following a 48 h seed imbibition with harpin or flg22 and then 7 d of growth, post inoculation. Significance of means was determined using a Student *t*-test in comparison to the water control. *** indicates *p* ≤ 0.001.

Seven days post inoculation (dpi) with *Pythium*, seedlings imbibed in harpin showed more resistance (Figure 1a) to *P. aphanidermatum* infection. The seedling growth was also significantly enhanced by harpin, with about 2.5-fold more total seedling length than that of water control and flg22 (Figure 1b). Flg22 imbibition did not improve seedling growth or trigger defense mechanisms that convey resistance to *P. aphanidermatum*. The results suggest that harpin and flg22 may induce different innate immune signaling pathways in cannabis.

### 3.2. Harpin and Flg22 Upregulate Certain Expression of Ethylene-Responsive Genes and Defense Genes

**Figure 2:**
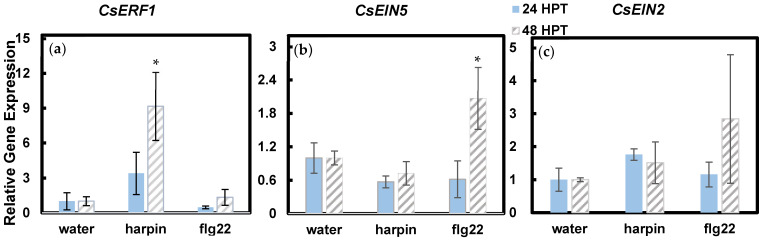
Effects of harpin and flg22 on the expression of ethylene responsive genes, *CsERF1* (**a**), *CsEIN5* (**b**), and *CsEIN2* (**c**), in cannabis seedlings. Cannabis seedings were submerged in water, harpin, or flg22 solutions for 24 h, then incubated on plates for 24 or 48 h. Statistically significant difference in comparison to water control was determined using Student *t*-test. * indicates *p* ≤ 0.05.

Both harpin and flg22 have been shown to be involved in ethylene-associated defense responses [2,3,17,18,19,21]. We examined how harpin and flg22 induce ethylene-responsive and defense-related genes. Three ethylene-regulated genes, *CsERF1*, *CsEIN5*, and *CsEIN2*, were chosen for the analysis. These genes are involved in plant development and pathogen/insect defense responses [2,3,16] and were upregulated in plants in response to harpin or flg22 treatment [2,3,17,18]. Figure 2 shows that harpin and flg22 induced different ethylene responsive genes. Harpin treated seedlings showed significant increase in *CsERF1* expression 48 h post treatment (HPT), while flg22 treatment did not affect *CsERF1* expression (Figure 2a). *CsEIN5* expression was only upregulated 48 h after flg22 treatment, while it did not respond to harpin treatment (Figure 2b). Neither treatment altered *CsEIN2* expression (Figure 2c).

**Figure 3:**
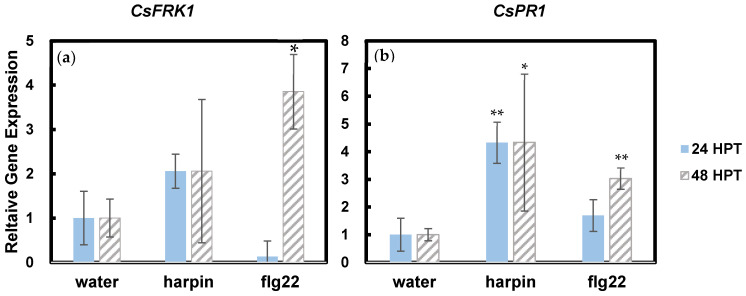
Effects of harpin and flg22 on the expression of defense marker genes, *CsFRK1* (**a**) and *CsPR1* (**b**), in cannabis seedlings. The RNA samples were the same as those shown in Figure 2. Statistically significant difference in comparison to water control was determined using Student *t*-test. * indicates *p* ≤ 0.05. ** indicates *p* ≤ 0.01.

We further examined two defense-related genes, *CsFRK1* and *CsPR1* [19,21]. Increased expression of both genes by flg22 has been reported [19,21]. In our assay, it is shown that flg22 significantly increased *CsFRK1* expression 48 h after treatment (Figure 3a). Although there was 2-fold increase 24 h after harpin treatment, the increase was not significant (Figure 3a). Harpin significantly upregulated *CsPR1* expression 24 h and 48 h after treatment, while flg22 increased *CsPR1* expression 48 h after treatment (Figure 3b). *PR1* is a marker gene for the salicylic acid (SA) signaling pathway, and the results suggest that both PAMPs can activate SA signaling and harpin may trigger the response faster than flg22 in cannabis. These results indicate that both harpin and flg22 can induce innate immune signaling in hemp and may enhance resistance to pathogens.

### 3.3. Both Harpin and Flg22 Promote Hemp Seedling Growth

**Figure 4:**
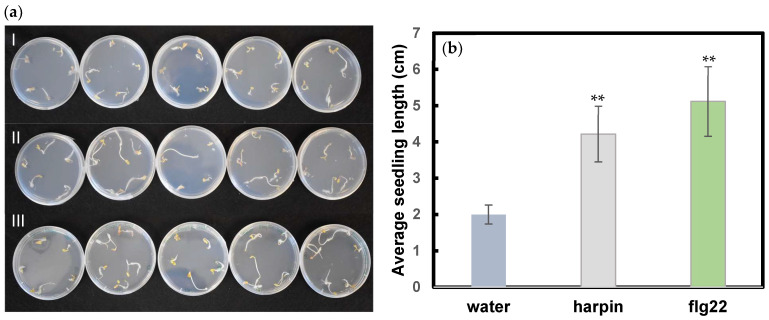
Enhancement of seedling growth by harpin and flg22. Experiments were conducted the same as shown in Figure 1 but without pathogen. (**a**) Growth of seedlings after 7 d on Petri plates with I, water agar, II, addition of harpin and III, addition of flg22. All five replicates for each treatment were shown. (**b**) Average seedling length. Statistically significant difference in comparison to water control was determined using Student *t*-test. ** indicates *p* ≤ 0.01.

Harpin has been commercialized as a plant growth and health stimulant [2,27,28]. Here, we examined the effects of both harpin and flg22 on hemp seedling growth. Compared to the water control, both harpin and flg22 treated seedlings showed more robust growth (Figure 4a). In addition, harpin showed a 2-fold and flg22 showed a 2.5-fold longer total seedling length than the control seedlings (Figure 4b). The results indicate that both harpin and flg22 pretreatments can stimulate hemp growth, which may be due to the activation of plant hormone signaling to enhance growth.

## 4. Discussion

*P. aphanidermatum* has been reported to cause crown and root rot in cannabis [29,30]. Research is lacking on the disease control of *P. aphanidermatum* in cannabis. Harpin and flg22 are well-studied bacterial PAMPs. They have been shown to trigger plant defense responses and promote plant growth [3,31,32]. We examined the effectiveness of harpin and flg22 on the induction of resistance to *P. aphanidermatum* and the stimulation of plant growth in cannabis seedlings. Harpin was effective in the resistance to *P. aphanidermatum* and it enhanced plant growth during infection, while flg22 pretreatment failed to induce defense responses and promote seedling growth (Figure 1b). It is known that harpin protein, Hpa1, from the bacterial blight pathogen found in rice acts similarly to the one from *E*. *amylovora*, indicating conserved effects among harpin proteins. It is possible that the defense mechanism induced by flg22 is not efficacious for protection from *P. aphanidermatum* infection.

Previous studies showed that flg22, but not harpin, induced jasmonate (JA) accumulation in *Vitis rupestris* [33]. Defense signaling pathways induced by harpin involve SA, which leads to systemic acquired resistance [34]. JA and SA signaling pathways have shown synergistic properties [35]. Flg22 activates PTI, while harpin triggers immunity like Effector-Triggered Immunity (ETI) [36]. Our results showed that the PTI marker gene, *CsFRK1*, was highly induced 48 h after flg22 treatment. Although harpin increased *CsFRK1*, there was no significant difference from the control (Figure 3a). *PR1* is a marker gene in the SA-induced signaling pathway [37]. In Arabidopsis, flg22 was also found to increase SA accumulation [38,39]. Both harpin and flg22 induced *CsPR1* expression, and therefore, activated SA signaling in cannabis seedlings, and harpin showed a faster induction than flg22 (Figure 3b).

Following harpin and flg22 imbibition, hemp seedlings showed similar activation of ethylene signaling as what has been reported in other plant species. However, cannabis seedlings responded differently to harpin and flg22. While neither harpin nor flg22 induced *CsEIN2* expression, harpin and flg22 activated different ethylene responsive genes examined in this report (Figure 2). In wheat, harpin only induced *EIN2* expression during aphid feeding [40,41]. It is possible that harpin and flg22 would not induce *CsEIN2* in cannabis under normal growth conditions. In addition, being an early signal molecule in ethylene signaling pathway, *CsEIN2* may be induced at earlier time points than 24 hpt.

There are plenty of management options to prevent pathogens in cannabis, such as *Botrytis cinerea*, which causes damping off and bud rot, or *Golovinomyces* spp., which causes powdery mildew [42]. Most recommended methods involve prevention or disposal of the plant. There is no published study as of now on the molecular mechanisms of pathogen defense responses in cannabis.

Our results provide scientific evidence that harpin and flg22 may activate different signaling molecules and induce defense responses in cannabis. Harpin is more effective than flg22 in triggering resistance to *P. aphanidermatum* in cannabis seedlings. Our findings provide insights to the disease control strategies of *P. aphanidermatum* and potentially other pathogens. Further studies on the effects of harpin on cannabis growth and cannabinoid and/or terpene biosynthesis may be explored.

## Figures and Tables

**Table 1:** Summary of homologs of cannabis genes used in qPCR.

Gene	Function	PAMP	Reference
Ethylene Response Factor 1 [ERF1]	Transcriptional activator which acts downstream of the ethylene signaling pathway. It mediates development and regulates pathogen defense genes [16].	harpin and flg22	[2,17]
Ethylene Insensitive Protein 5 [EIN5]	Required for plant growth enhancement [2]. Acts downstream of CTR1, a negative regulator of the pathway. If ethylene is present, CTR1 does not act, and the pathway can be activated [3].	harpin	[3]
Ethylene Insensitive Protein 2 [EIN2]	Required for insect resistance in plants [2]. Protein located downstream of CTR1 in the ethylene pathway. If CTR1 is inhibited, then EIN2 may be activated. It is a Nramp-like protein, meaning it is a natural resistance-associated macrophage protein [18].	harpin	[2,18]
Flg22-Induced Receptor Like Kinase 1 [FRK1]	Associated with early defense signaling as well as mediating senescence. Following flg22 exposure in an organism, FRK1 regulation likely increases accordingly [19].	flg22	[19]
Pathogenesis Related Protein 1 [PR1]	Gene expression increases following pathogen detection [20]. It is an indicator of systemic acquired resistance.	flg22	[21]

## Data Availability

Not applicable.

## Supplementary Materials

The following are available online at: https://www.mdpi.com/article/10.3390/plants11091178/s1, Figure S1: Genetic examination of hemp strain Finola2; Figure S2: Display of seed placement; Table S1: Primers used in qPCR.

## Abbreviations

PAMP	Pathogen-Associated Molecular Pattern
PRR	Pattern Recognition Receptors
FLG22	Flagellin 22
PTI	PAMP-Triggered Immunity
ERF1	Ethylene Response Factor 1
EIN5	Ethylene Insensitive Protein 5
EIN2	Ethylene Insensitive Protein 2
FRK1	Flagellin22-induced Receptor-like Kinase 1
PR1	Pathogenesis-Related Protein 1
ETI	Effector-Triggered Immunity
JA	Jasmonic acid
SA	Salicylic Acid
