# Supplementary material for: Effects of Harpin and Flg22 on Growth Enhancement and Pathogen Defense in Cannabis sativa Seedlings
Source: Plants (Basel). 2022 Apr 27;11(9):1178. doi: 10.3390/plants11091178 (PMC9101757; doi:10.3390/plants11091178)
Supplement: Supplementary file 1 [file plants-11-01178-s001.zip › plants-1660551-supplementary.pdf]

Supplementary Material

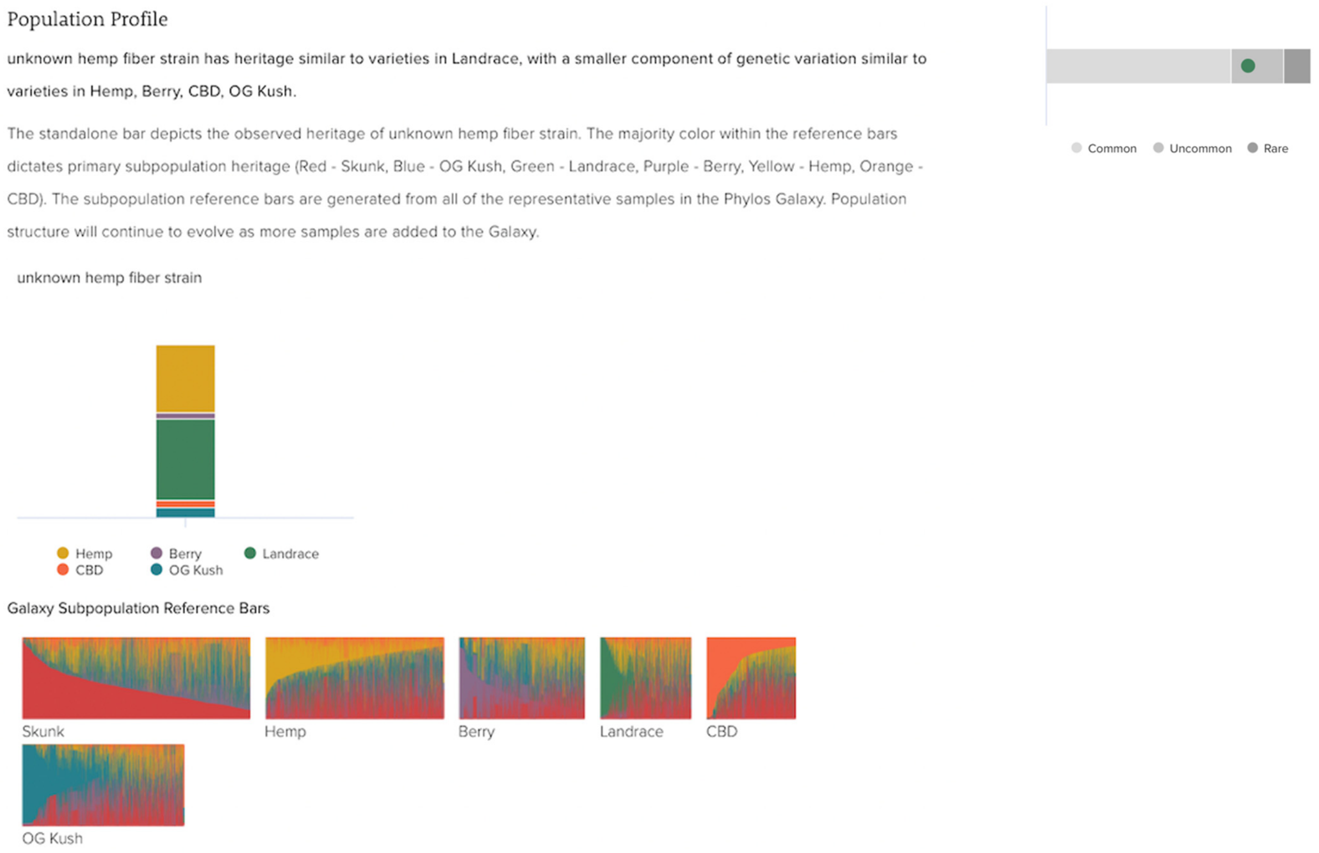

Figure S1. Genetic examination of the hemp strain used in this report by Phylos. The genetic information of this strain is not in their database and the closest variety is Finola. We named this strain "Finola2". Genetic information Obtained through sending plant material to Phylos galaxy in Washington, USA.

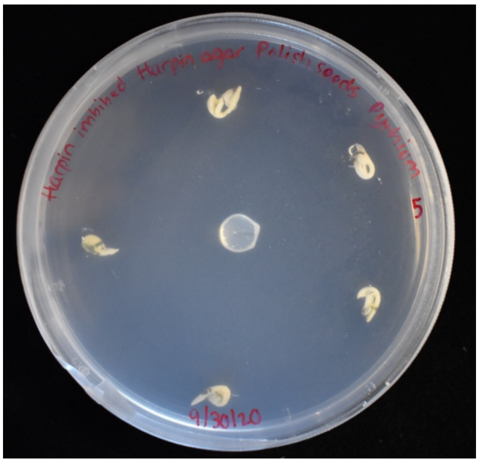

Figure S2. Display of seed placement on the agar plate. Seed coats were removed and placed equidistantly from the edge of the Petri plate and from the mycelial plug containing *Pythium aphanidermatum* in the center of the plate. For Figure 4, no plug was added.

Table S1. Primers used in qPCR. All primers for *Cannabis sativa* were adapted as homologs from the *Arabidopsis thaliana* genome using NCBI and IDTDNA.

Gene	Forward Primer	Reverse Primer
CsUbiquitin	GCCAGGATGGCAATGAAGTA	GCTATAGAGTTGAACTCCACAG
CsERF1	GAAGTCGTACCGAGGCGTTC	TCCACGCATCGAAAAAGCAG
CsEIN5	TCTCTCGACAACCGCTGTAA	TCCATTCATCCCTTCACTAATTTCT
CsEIN2	ACCAGCATCAGCACAACAGA	AACTGCGGTGGTGCATTTTC
CsFRK1	CCTCAAGTGGGTTGACTGGG	TCGCTTGGAACTGAACCGAT
CsPR1	CGGAGAAGGCTGACTACGAC	TAGCACACCCAAGACGAACC
